# Structure–function insights into the initial step of DNA integration by a CRISPR–Cas–Transposon complex

**DOI:** 10.1038/s41422-019-0272-2

**Published:** 2020-01-10

**Authors:** Ning Jia, Wei Xie, M. Jason de la Cruz, Edward T. Eng, Dinshaw J. Patel

**Affiliations:** 1 Structural Biology Program, Memorial Sloan Kettering Cancer Center, New York, NY 10065 USA; 2 Simons Electron Microscopy Center, New York Structural Biology Center, New York, NY 10027 USA

**Keywords:** Cryoelectron microscopy, Transposition

Dear Editor,

CRISPR (clustered regularly interspaced short palindromic repeats)–Cas (CRISPR-associated genes) surveillance complexes are RNA-based adaptive immune systems employed by prokaryotes against invading nucleic acids from bacteriophages and plasmids.^1,2^ The CRISPR-derived RNAs (crRNAs) guide the Cas effector complex to target and degrade the invading nucleic acids. Recently, bioinformatics analyses have revealed the presence of CRISPR–Cas loci in bacterial Tn7-like transposons, thereby implicating a functional relationship between RNA-guided DNA targeting and transposition, with the latter representing a new role unrelated to host defense.^3^ Support for this concept has emerged from recent functional studies on type I-F and type V-K effectors involved in sequence-specific DNA transposition,^4,5^ thereby significantly broadening the potential biological applications of CRISPR–Cas technology. To complement the available functional studies, our efforts have focused on structural studies of the *Vibrio cholerae* Tn6677 multi-subunit type I-F Cascade^crRNA^–TniQ complex, whereby transposition subunit TniQ initiates DNA transposition with the eventual help of other transposition-associated proteins TnsA, TnsB and TnsC in the gene cluster (Fig. 1a).

**Fig. 1:**
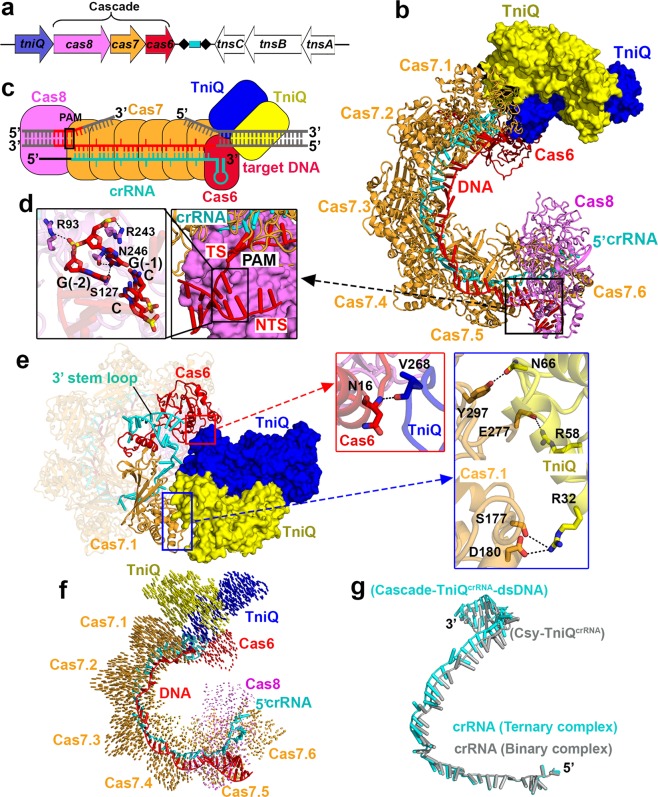
Cryo-EM structures of type I-F Cascade^crRNA^–TniQ complexes. **a** The *cas* and transposition-related genes in the *Vibrio cholerae* Tn6677 type I-F CRISPR–transposon system. **b**, **c** Schematic (**c**) and ribbon (**b**) representations of cryo-EM structure of Cascade^crRNA^–TniQ–dsDNA ternary complex. **d** PAM recognition by Cas8. Detailed interactions are shown in the expanded panels. **e** Interactions between the individual transposition protein TniQ and Cas6 and Cas7.1. Inset panels pointed by arrows provide detailed interactions. **f** Structure comparison between Cascade^crRNA^–TniQ binary complex and Cascade^crRNA^–TniQ–dsDNA ternary complex based on alignment of the Cas8 subunit. Vector length correlates with the domain movement scale. **g** Superposition of crRNA in the binary (in silver) and ternary (in cyan) complexes.

Here we report cryo-EM structures of a *V. cholerae* type I-F Cascade^crRNA^ in complex with transposition subunit TniQ before (binary Cascade^crRNA^–TniQ) and after (ternary Cascade^crRNA^–TniQ–dsDNA) target double-stranded DNA (dsDNA) binding at an average resolution of 2.9 Å and 3.2 Å, respectively (Supplementary information, Figs. S1 and  2). Cas6 and TniQ can be readily traced in the 2.9 Å structure of the binary complex, thereby providing insights into how the three Cas subunits (Cas8, Cas7, and Cas6) of the multi-subunit type I-F Cascade are assembled in an intertwined helical topology with crRNA and TniQ.

The binary complex reveals a 1:6:1 Cas8:Cas7:Cas6 subunit stoichiometry similar to that reported for type I-F Cascade^crRNA^ complexes,^6–8^ but contains a head-to-tail aligned TinQ dimer, whose individual monomers are bound to Cas7.1 and Cas6, so as to facilitate the first step of DNA transposition (Supplementary information, Fig. S3a). The complex is assembled with a 60-nucleotide (nt) crRNA processed by the CRISPR-specific endoribonuclease Cas6 from long precursor CRISPR transcripts (pre-crRNA), which contains 28-nt repeat sequences separated by 32-nt plasmid- or phage-derived spacer sequences. The partially palindromic repeat sequence leads to a stable stem-loop structure that could be recognized and cleaved by Cas6 (Supplementary information, Fig. S3b). Cas6 binds to the 3′ stem-loop of crRNA, while forming multiple polar interactions with Cas7.1 (Supplementary information, Fig. S3b, left insert). It has been shown that the trimming of pre-crRNA into crRNA is essential for both complex assembly^9^ and DNA transposition, given that the H29A mutant located in the pre-crRNA cleavage pocket shows no DNA transposition activity.^5^ Following Cas6-mediated maturation of crRNA, Cas proteins are assembled with crRNA, with the 5′ end recognized by Cas8 and the 3′ stem-loop held by Cas6, which is kinked by Cas7 thumb motifs in a periodic “5+1” pattern (Supplementary information, Fig. S3c).

Our Cascade^crRNA^–TniQ–dsDNA ternary complex contains a G–G/C–C PAM (protospacer adjacent motif) sequence (Fig. 1b), which is required for binding of the target sequence, allowing CRISPR–Cas systems to discriminate between self and non-self.^10^ The target sequence contains 12-base pair (bp) of PAM-proximal duplex DNA and 51-bp of PAM-distal duplex DNA (Fig. 1c; Supplementary information, Fig. S4). We could observe only 5-bp of PAM-proximal duplex DNA with 2-nt overhang at the 3′ end of the non-target strand (NTS), and a traceable 32-nt target DNA strand complementary and paired to 32-nt spacer RNA, but no clear density for the PAM-distal duplex segment, which is adjacent to bound TniQ. The traceable parts of the crRNA–dsDNA are colored, while non-traceable parts are shown in grey (Fig. 1c; Supplementary information, Fig. S4). The G–G/C–C PAM is specifically recognized by Ser127 and Asn246 in Cas8 (Fig. 1d). Arg243 forms a wedge and stacks with PAM G (–1), facilitating the formation of crRNA–target DNA heteroduplex and displacing the NTS, thus leading to the onset of R-loop formation.

It has been proposed that the transposition protein TniQ serves as an important connection between sequence-specific DNA targeting by the Cascade^crRNA^ complex and DNA transposition by the accompanying transposase subunits TnsA, TnsB, and TnsC.^5^ In line with this concept, we observe that TniQ forms a head-to-tail dimer whose individual monomers simultaneously bind to Cas6 and Cas7.1 of the Cascade^crRNA^ complex (Fig. 1b, c), with clear density observed at the interface between TniQ and both Cas6 and Cas7.1. One TniQ interacts with Cas6 via a main chain polar interaction between Val268 and Asn16, whereas the other TniQ interacts with Cas7.1 by multiple polar interactions between side chains over a larger interface (Fig. 1e, inset). We also determined the crystal structure of apo-TniQ at 2.1 Å resolution, demonstrating that TniQ forms a head-to-tail dimer with a dimeric interface of 1931 Å^2^ (Supplementary information, Fig. S5a). The dimer result was confirmed by SEC-MALS in solution (Supplementary information, Fig. S5b). Superposition of the TniQ dimer in the cryo-EM structure of ternary Cascade^crRNA^–TniQ–dsDNA complex (in color) with the crystal structure of the apo-form (in grey) reveals minimal conformational changes with an RMSD of 1.19 Å; the loop interacting with Cas6 becomes ordered and the helix interacting with Cas7.1 undergoes slight movement (circled in green, Supplementary information, Fig. S5c).

Notably, the binary and DNA-bound ternary Cascade^crRNA^–TniQ complexes superpose with an RMSD of 2.33 Å. Minimal conformational changes are observed on ternary complex formation in the presence of bound target DNA, reflecting slight opening of the entire complex (Fig. 1f) and crRNA (Fig. 1g), especially within the TniQ-bound end.

Our studies, and a parallel contribution^11^ provide insights into DNA targeting by the Cascade^crRNA^–TniQ complex, which represents an essential initial step in crRNA-guided DNA integration. The studies also provide structural insights into the potential use of RNA-guided Tn7-like transposons for genome editing. Further studies are required to investigate how the Cascade^crRNA^–TniQ complex recruits other transposon-associated proteins (TnsA, TnsB, and TnsC) to facilitate DNA transposition.

The atomic coordinates and EM maps have been deposited into the Protein Data Bank with the accession numbers 6V9P, 6V9Q, and 6VBW, and the EM Data Bank with the accession numbers EMD-21126 and EMD-21146.

## Supplementary information

Supplementary information, Figures and Tables
