# Supplementary material for: Structure–function insights into the initial step of DNA integration by a CRISPR–Cas–Transposon complex
Source: Cell Res. 2020 Jan 10;30(2):182–4. doi: 10.1038/s41422-019-0272-2 (PMC7015049; doi:10.1038/s41422-019-0272-2)
Supplement: Supplementary file 1 — Supplementary information, Figures and Tables [file 41422_2019_272_MOESM1_ESM.pdf]

## Materials and Methods

### Protein Expression and Purification

The full-length *Vibrio cholerae Tn6677 cas* genes, cas8, cas7, cas6 and the transposition-associated *tniQ* genes were synthesized and cloned into different expression vectors. *cas8* and *tniQ* genes were subcloned into a modified pRSF-Duet-1 vector (Novagen), in which tni*Q* gene was attached with N-terminal His6-SUMO tag following an ubiquitin-like protease (ULP1). cas7 were cloned into pET-Duet-1. *cas6* gene and the synthetic CRISPR gene were cloned into pCDFDuet-1. All these vectors were co-transformed into *Escherichia coli* BL21 (DE3) strain by induction with 0.25 mM isopropyl-β-D-1-thiogalactopyranoside (GoldBio) at 16 °C for 20 hr. Cells were harvested by centrifugation and resuspended in lysis buffer (20 mM Tris-HCl, pH 8.0, 500 mM NaCl, 5% glycerol, 20 mM imidazole, 7 mM β-mercaptoethanol). The harvested cells were then lysed by the EmulsiFlex-C3 homogenizer (Avestin) and centrifuged at 20,000 rpm for 30 min in a JA-20 fixed angle rotor (Avanti J-E series centrifuge, Beckman Coulter). The supernatant was applied to 5 mL HisTrap Fast flow column (GE Healthcare). The protein was eluted with lysis buffer supplemented with 500 mM imidazole after washing the column with 10 column volumes of lysis buffer and 2 column volumes of lysis buffer supplemented with 40 mM imidazole. The elution fractions were further dialyzed against buffer A (20 mM Tris-HCl, pH 8.0, 100 mM NaCl, 5% glycerol, 7mM β-mercaptoethanol), and applied on 5 mL HiTrap Q Fast flow column (GE Healthcare). Proteins were eluted by a linear gradient from 100 mM to 1 M NaCl in 20 column volumes, and then concentrated in 50 kDa molecular mass cut-off concentrators (Amicon) before further purification over a Superdex 200 increase 10/300 GL column (GE Healthcare) pre-equilibrated in buffer B (20 mM HEPES, pH 7.5, 150 mM NaCl, 2 mM DTT).

Apo-*tniQ* was cloned into pRSF-Duet-1 vector (Novagen), in which *tniQ* gene was attached with N-terminal His6-SUMO tag following an ubiquitin-like protease (ULP1). And Apo-TniQ was purified by the same method as that for Cascade$^{crRNA}$-TniQ complex.

### Crystallization, Data Collection, and Structure Determination

Crystals of apo-TniQ were also grown at 20 °C using hanging-drop vapor diffusion by mixing 1 μl protein solution with 1 μl reservoir solution containing 2% PEG 6K, 0.1 Bicine pH 9.0. The crystals were cryoprotected using the reservoir solution supplemented with 30% glycerol. Data were collected at 100 K at the Advanced Photo Source (APS) at the Argonne National Laboratory. The diffraction data were processed with the NECAT RAPD online server.

The apo-TniQ structure was solved by molecular replacement method in Phenix[1], using raw TniQ model built from cryo-EM map of Cascade$^{rRNA}$-TniQ binary complex. The refinement was performed with Phenix[1] and COOT[2]. The statistics of the diffraction data are summarized in Supplementary information, Table S1. All structure figures were prepared with PyMOL (http://www.pymol.org/).

**Cryo-EM Sample Preparation and Data Acquisition**

3.0 µl of ~0.5 mg/ml purified Cascade$^{crRNA}$-TniQ binary and Cascade$^{crRNA}$-TniQ-dsDNA ternary complexes were applied onto glow-discharged UltrAuFoil 300 mesh R1.2/1.3 grids (Quantifoil), respectively. Grids were blotted for 2s at ~100% humidity and flash frozen in liquid ethane using an FEI Vitrobot Mark IV. For the Cascade$^{crRNA}$-TniQ binary complex, images were collected on FEI Titan Krios electron microscope operated at an acceleration voltage of 300 kV with a Gatan K3 Summit detector with a 1.08 Å pixel size at the National Cancer Institute's National Cryo-EM Facility. The defocus range was set from -1.0 µm to 2.5 µm. Movies were recorded in super-resolution mode at a dose rate of 14.7 $e^-/Å^2/s$ with a total exposure time of 3.4 s, for an accumulated dose of 50 $e^-/Å^2$. Intermediate frames were recorded every 0.068 s for a total number of 50 frames. For the Cascade$^{crRNA}$-TniQ-dsDNA ternary complex, images were collected on FEI Titan Krios electron microscope operated at an acceleration voltage of 300 kV with a Gatan K3 Summit detector with a 1.1 Å pixel size at Memorial Sloan Kettering Cancer Center. The defocus range was set from -1.0 µm to 2.5 µm. Movies were recorded in super-resolution mode at a dose rate of 16.4 $e^-/Å^2/s$ with a total exposure time of 3 s, for an accumulated dose of 49.3 $e^-/Å^2$. Intermediate frames were recorded every 0.075 s for a total number of 40 frames.

**Image Processing**

For Cascade$^{crRNA}$-TniQ binary dataset, motion correction was performed with MotionCor2[3]. Contrast transfer function parameters were estimated by Ctffind4[4]. All other steps of image processing were performed by RELION 3[5]. Templates for automated particle selection were generated from 2D-averages of ~2,000 manually picked particles. Automated particle selection resulted in 3,134,195 particles from 3,614 images. After two rounds of 2D classification, a total of 1,423,694 particles were selected for 3D classification using the initial model generated by RELION as reference. Particles corresponding to the best class with the highest-resolution features were selected and subjected to the second round of 3D classification. One of 3D classes with good secondary structural features and the corresponding 134,856 particles were polished using RELION particle polishing, yielding an electron microscopy map with a resolution of 2.9 Å after 3D auto-refinement (Supplementary information, Fig. S1).

The dataset for Cascade$^{crRNA}$-TniQ-dsDNA ternary complex was processed by the same procedure as above. Briefly, 3,036,464 particles were autopicked from 5,271 images, 55,900 particles were selected for the final 3D reconstruction after two rounds of 2D and 3D classification, resulting in a Cascade$^{crRNA}$-TniQ-dsDNA ternary complex map with an overall resolution of 3.2 Å (Supplementary information, Fig. S2).

All resolutions were estimated using RELION 'post-processing' by applying a soft mask around the protein density and the Fourier shell correlation (FSC) = 0.143 criterion. Local resolution estimates were calculated from two half data maps using ResMap[6]. Further details related to data processing and refinement are summarized in Supplementary information, Table S2.

**Atomic Model Building and Refinement**

For the Cascade$^{crRNA}$-TniQ binary complex, the initial models of Cas8, Cas7, Cas6 and TniQ were manually built in COOT based on the bulky side chains to register the sequence[2]. All models were refined against summed maps using phenix.real_space_refine[1] by applying geometric and secondary structure restraints. For Cascade$^{crRNA}$-TniQ-dsDNA ternary complex, the structure of Cascade$^{crRNA}$-TniQ binary complex was docked into the cryo-EM density map using UCSF Chimera[7] and then manually rebuilt in COOT[2]. Density for TniQ subunits is not good enough for tracing all the residues, we then docked the model obtained from crystal structure of TniQ. All models were refined against summed maps using phenix.real_space_refine[1] by applying geometric and secondary structure restraints. All figures were prepared by PyMol (http://www.pymol.org) or Chimera[7]. The statistics for data collection and model refinement are shown in Supplementary information, Table S2.

**SEC-MALS Experiments**

For protein molar mass determination, purified TniQ proteins were analyzed using an ÄKTA-MALS system. A mini DAWN TREOS multi-angle light scattering detector (Wyatt Technology) and an Optilab T-rEX refractometer (Wyatt Technology) were used in-line with Superdex200 10/300 gel filtration column (GE Healthcare) pre-equilibrated in buffer B at a flow rate of 0.2 mL/min. Separation and ultraviolet detection were performed by ÄKTA Pure system (GE Healthcare), light scattering was monitored by miniDAWN TREOS system, and concentration was measured by the Optilab T-rEX differential refractometer. Molar masses of proteins were calculated using the Astra 6.1 program (Wyatt Technology) with a dn/dc value of 0.185 mL/g.

**References**

1 Adams PD, Afonine PV, Bunkoczi G *et al.* PHENIX: a comprehensive Python-based system for macromolecular structure solution. *Acta crystallographica Section D, Biological crystallography* 2010; **66**:213-221.
2 Emsley P, Lohkamp B, Scott WG, Cowtan K. Features and development of Coot. *Acta crystallographica Section D, Biological crystallography* 2010; **66**:486-501.
3 Zheng SQ, Palovcak E, Armache JP, Verba KA, Cheng Y, Agard DA. MotionCor2: anisotropic correction of beam-induced motion for improved cryo-electron microscopy. *Nature methods* 2017; **14**:331-332.
4 Rohou A, Grigorieff N. CTFFIND4: Fast and accurate defocus estimation from electron micrographs. *J Struct Biol* 2015; **192**:216-221.
5 Scheres SH. RELION: implementation of a Bayesian approach to cryo-EM structure determination. *J Struct Biol* 2012; **180**:519-530.
6 Kucukelbir A, Sigworth FJ, Tagare HD. Quantifying the local resolution of cryo-EM density maps. *Nature methods* 2014; **11**:63-65.

7 Pettersen EF, Goddard TD, Huang CC *et al.* UCSF Chimera--a visualization system for exploratory research and analysis. *J Comput Chem* 2004; **25**:1605-1612.

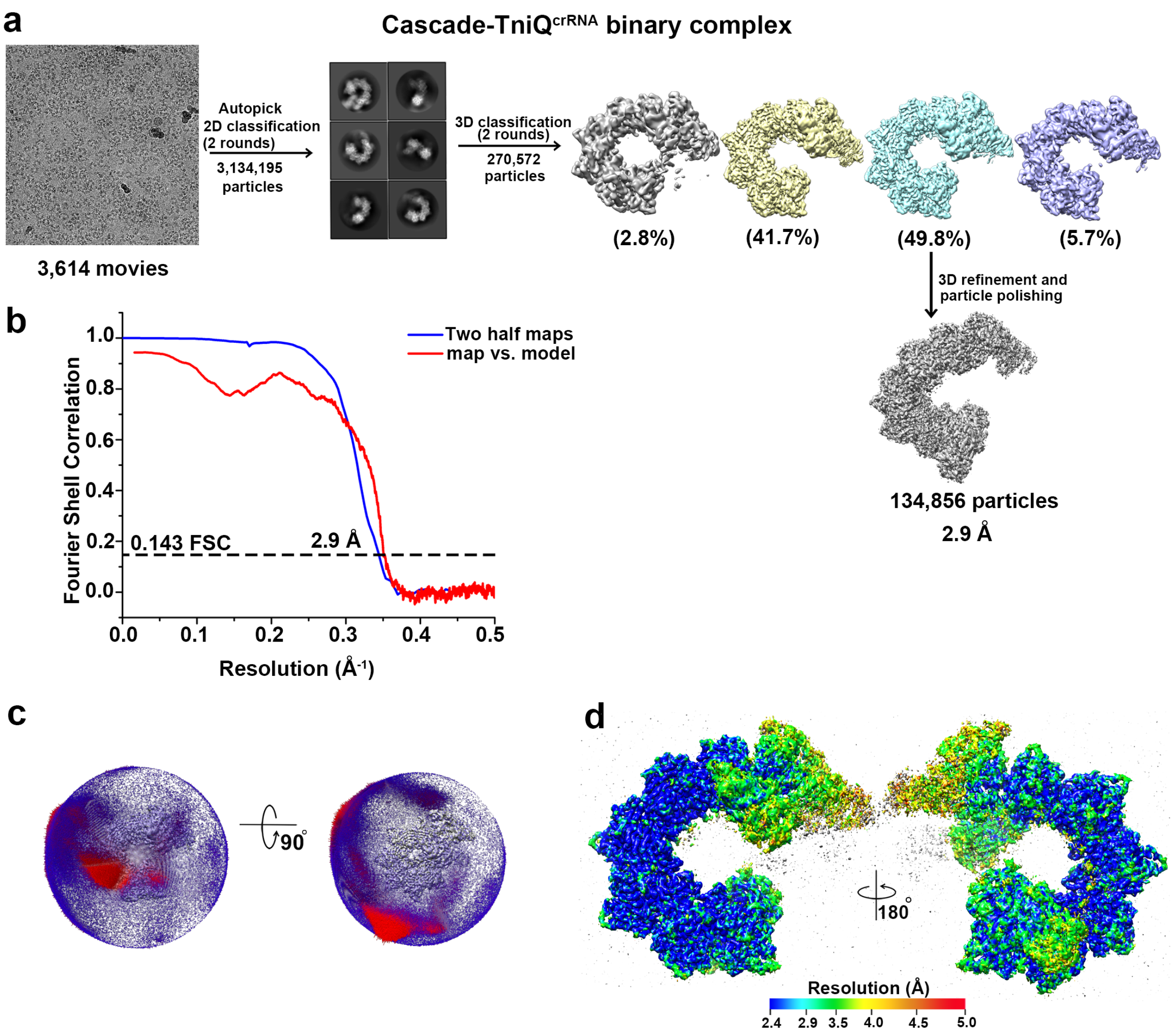


**Supplementary information, Fig. S1. Cryo-EM Reconstruction of Cascade$^{crRNA}$-TniQ binary complex. a** Flow chart of image processing of Cascade$^{crRNA}$-TniQ binary complex. **b** Fourier Shell Correlation (FSC) curve of Cascade$^{crRNA}$-TniQ binary complex and between two half maps that were calculated from two half datasets, and between the cryo-EM map and corresponding model. **c** Euler angle distribution of cryo-EM particles for calculating the final EM map of Cascade$^{crRNA}$-TniQ binary complex. The position of each sphere relative to the density map (gray in the center) corresponds to its angular assignment, with the radius of sphere proportional to the number of particles in that orientation. **d** Final 3D reconstructed map of Cascade$^{crRNA}$-TniQ binary complex colored according to local resolution. The majority of the map is higher than 3 Å, with relatively poor density on both ends of the map.

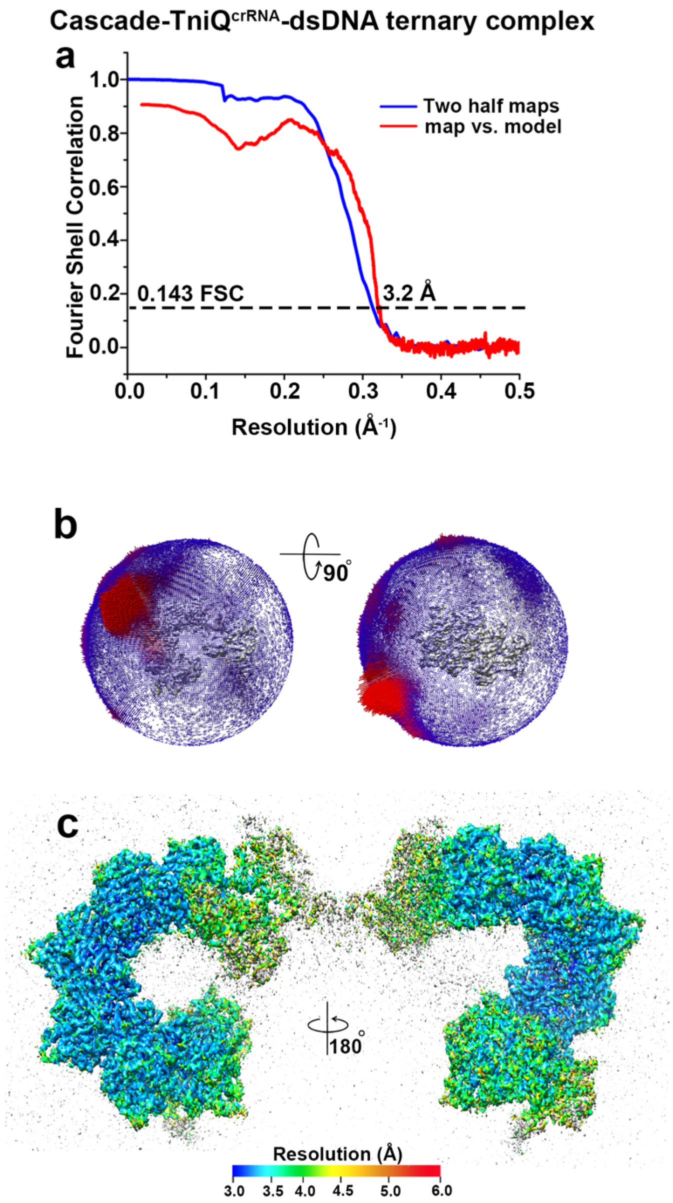


**Supplementary information, Fig. S2. Cryo-EM Reconstruction of Cascade$^{crRNA}$-TniQ-dsDNA ternary complex. a** Fourier Shell Correlation (FSC) curve of Cascade$^{crRNA}$-TniQ-dsDNA complex and between two half maps that were calculated from two half datasets, and between the cryo-EM map and corresponding model. **b** Euler angle distribution of cryo-EM particles for calculating the final EM map of Cascade$^{crRNA}$-TniQ-dsDNA ternary complex. The position of each sphere relative to the density map (gray in the center) corresponds to its angular assignment, with the radius of sphere proportional to the number of particles in that orientation. **c** Final 3D reconstructed map of Cascade$^{crRNA}$-TniQ-dsDNA ternary complex colored according to local resolution.

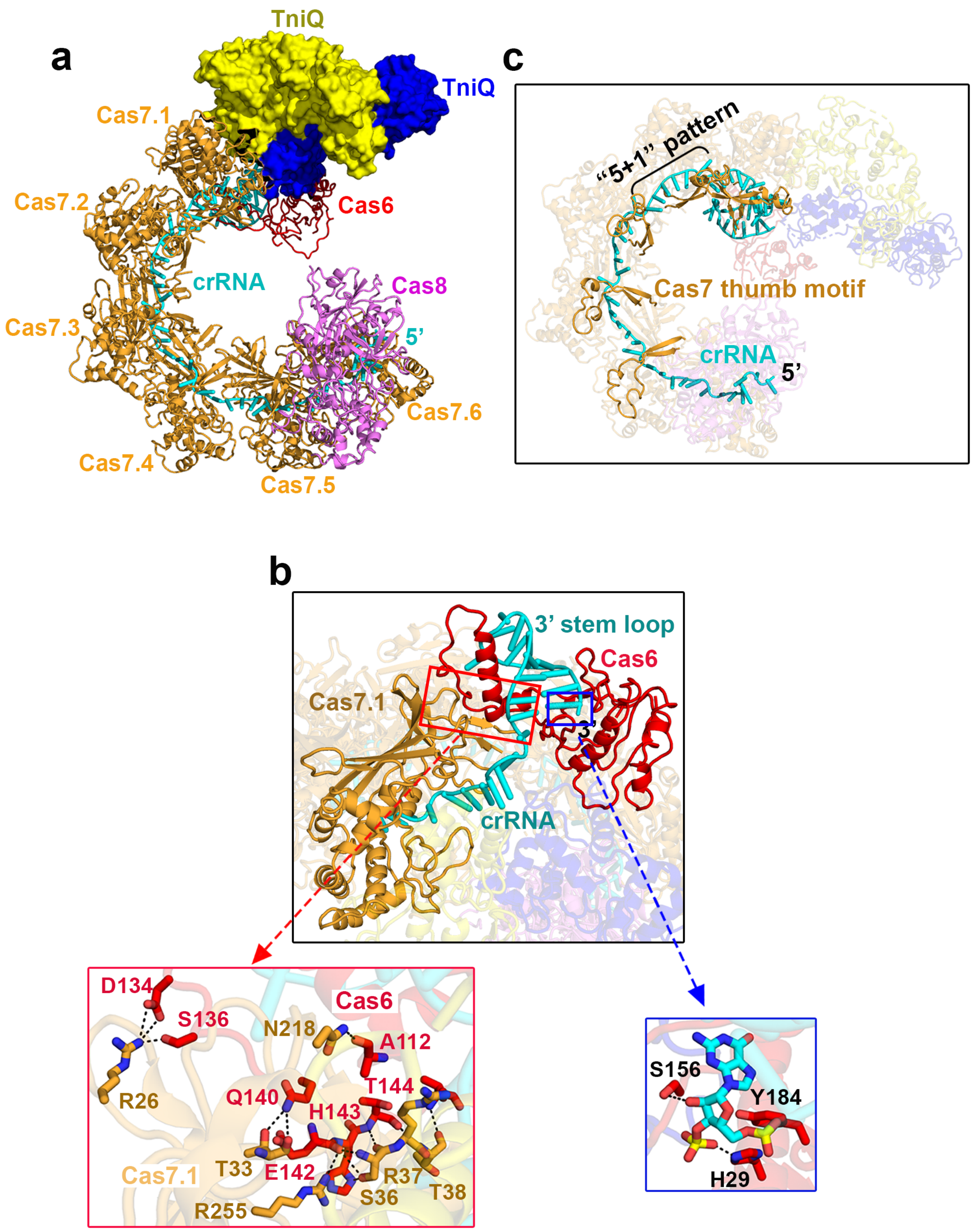


**Supplementary information, Fig. S3. cryo-EM structures of Cascade$^{crRNA}$-TniQ binary complex. a** Ribbon representation of cryo-EM structure of Cascade$^{crRNA}$-TniQ binary complex. **b** Interactions between Cas6 and Cas7.1 (left insert), and key residues involved in cleavage of 3' stem loop of crRNA by Cas6 (right insert). Previous reports show that mutation of either of

His29, Ser150 or Tyr176 in the RNA cleavage pocket of *Pseudomonas aeruginosa* Cas6f (corresponding to His29, Ser156 and Tyr184 in Cas6) leads to defects in RNA cleavage[1]. It was proposed that His29 (His29 in Cas6) deprotonates the 2-hydroxyl nucleophile held in place by Ser148 (Ser156 in Cas6), leading to attack on the scissile phosphate for RNA cleavage. **c** Cas7 thumb motifs kinks crRNA in a'5+1' pattern.

1 Haurwitz, R. E., Sternberg, S. H. & Doudna, J. A. The EMBO journal 31, 2824-2832, (2012).

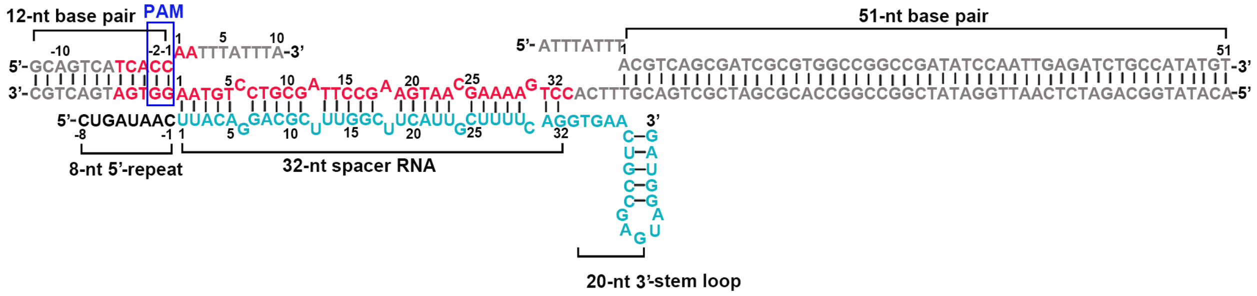


**Supplementary information, Fig. S4. Schematic drawing for the sequences of crRNA and target DNA.** The traceable parts of the crRNA-dsDNA are colored, while non-traceable parts are shown in grey.

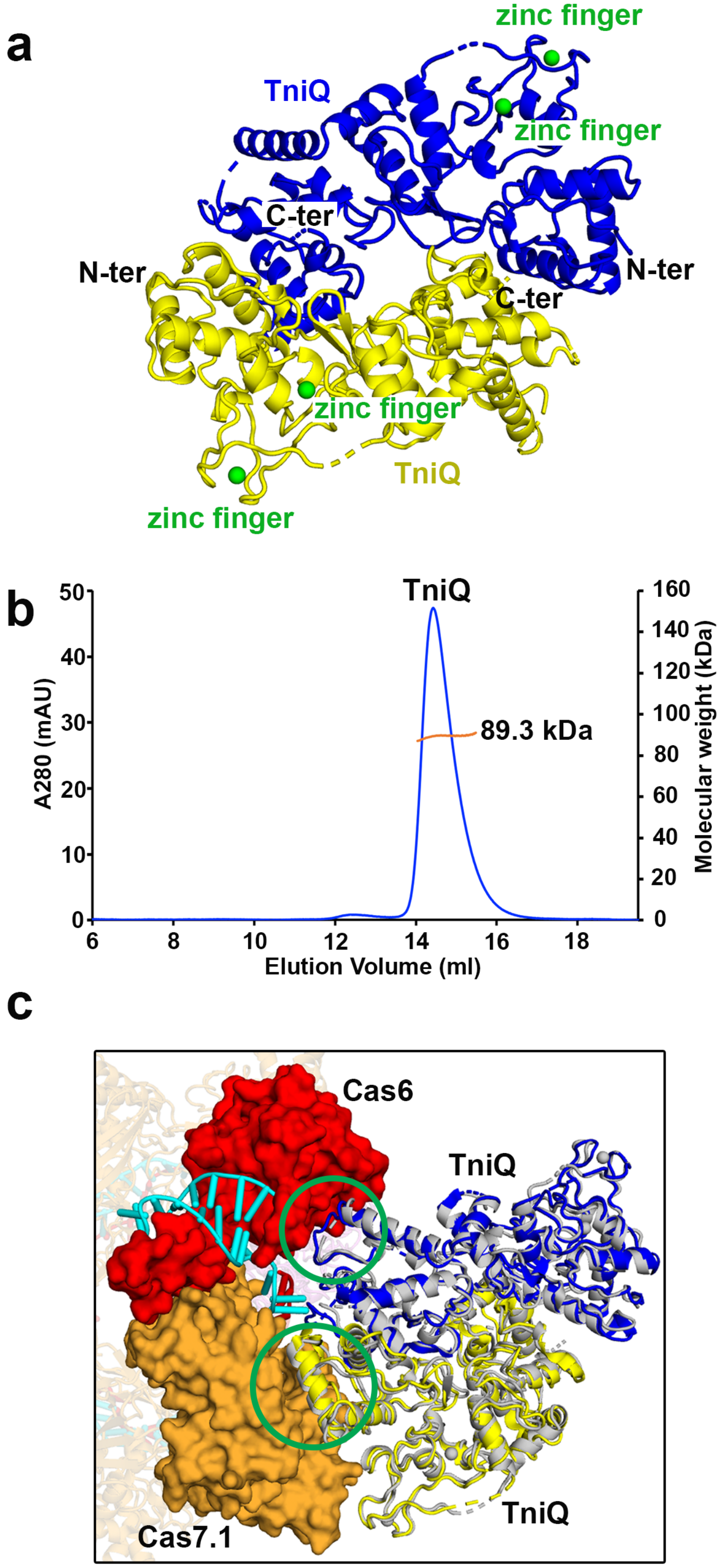


**Supplementary information, Fig. S5. The transposition protein TniQ folds as a head-to-tail dimer.** **a** Crystal structure of apo-TniQ as a head-to-tail dimer. **b** SEC-MALS detection of

oligomeric states of TniQ in solution. The horizontal orange line represents the calculated molecular mass (89.3 kDa) for dimer, which is close to its theoretical molecular mass of dimer (91.8 kDa). **c** Superposition of apo-TniQ (in grey) and TniQ in Cascade$^{crRNA}$-TniQ-dsDNA ternary complex (in color). Interactions between individual TniQ with Cas6 and Cas7.1 are circled in green.

**Supplementary information, Table S1. X-ray Statistics for Data collection and model refinement**

| | TniQ |
|---|---|
| **Data Collection** | |
| Beam line | APS-ID24C |
| Wavelength (Å) | 0.9792 |
| Space group | $P4_12_12$ |
| Cell parameters | |
| a, b, c (Å) | 69.82, 69.82, 179.49 |
| α, β, γ (°) | 90.00, 90.00, 120.00 |
| Resolution (Å) | 50.00-1.93 (1.97-1.93)[a] |
| $R_{pim}$ (%) | 1.1 (69.1) |
| I/ σ (I) | 34.7 (1.0) |
| Completeness (%) | 99.9 (99.0) |
| CC (1/2) | 1.00 (0.78) |
| Average Redundancy | 24.4 (11.7) |
| No. of unique reflections | 34,495 (2,240) |
| **Refinement** | |
| $R_{work}/R_{free}$ (%) | 23.42/26.64 |
| Protein residues | 374 |
| Average B factors ($Å^2$) | 60.18 |
| R.m.s. deviations | |
| Bond lengths (Å) | 0.008 |
| Bond angles (°) | 1.24 |
| Ramachandran plot (%) | |
| Favored | 95.34 |
| Allowed | 4.66 |
| Outliers | 0 |

[a] Highest resolution shell (in Å) shown in parentheses.

**Supplementary information, Table S2. Cryo-EM Statistics for Data collection and model refinement**

| | **Cascade$^{crRNA}$-TniQ** | **Cascade$^{crRNA}$-TniQ-dsDNA** |
|---|---|---|
| **Data collection and processing** | | |
| Magnification | 22,500 | 22,500 |
| Voltage (kV) | 300 kV | 300 kV |
| Electron exposure ($e^{-}$/ Å$^2$) | 50 | 49 |
| Defocus rang (μm) | -1.0 to -2.5 | -1.0 to -2.5 |
| Pixel size (Å) | 1.08 | 1.10 |
| Initial particles (no.) | 3,134,195 | 3,036,464 |
| Final particles (no.) | 134,856 | 55,900 |
| Map resolution (Å) | 2.9 | 3.2 |
| FSC threshold | 0.143 | 0.143 |
| Map sharpening B factor (Å$^2$) | -64.28 | -69.21 |
| **Refinement** | | |
| Model resolution (Å) | 3.03 | 3.33 |
| FSC threshold | 0.5 | 0.5 |
| Model composition | | |
| Protein residues | 3,433 | 3,464 |
| Nonhydrogen atoms | 28,766 | 29,995 |
| B factors (Å$^2$) | 49.91 | |
| R.m.s. deviations | | |
| Bond lengths (Å) | 0.005 | 0.007 |
| Bond angles (°) | 1.028 | 0.954 |
| Validation | | |
| MolProbity score | 1.84 | 1.75 |
| Clashscore | 4.60 | 4.25 |
| Poor rotamers (%) | 0.33 | 0.29 |
| Ramachandran plot | | |
| Favored (%) | 91.30 | 91.20 |
| Allowed (%) | 8.60 | 8.60 |
| Disallowed (%) | 0.10 | 0.20 |
